# Microfluidics for long-term single-cell time-lapse microscopy: Advances and applications

**DOI:** 10.3389/fbioe.2022.968342

**Published:** 2022-10-12

**Authors:** Paige Allard, Fotini Papazotos, Laurent Potvin-Trottier

**Affiliations:** ^1^ Department of Biology, Concordia University, Montréal, QC, Canada; ^2^ Department of Physics, Concordia University, Montréal, QC, Canada; ^3^ Centre for Applied Synthetic Biology, Concordia University, Montréal, QC, Canada

**Keywords:** microfluidics, time-lapse microscopy, cell screening, single-cell analysis, phenotypic heterogeneity, cellular dynamics

## Abstract

Cells are inherently dynamic, whether they are responding to environmental conditions or simply at equilibrium, with biomolecules constantly being made and destroyed. Due to their small volumes, the chemical reactions inside cells are stochastic, such that genetically identical cells display heterogeneous behaviors and gene expression profiles. Studying these dynamic processes is challenging, but the development of microfluidic methods enabling the tracking of individual prokaryotic cells with microscopy over long time periods under controlled growth conditions has led to many discoveries. This review focuses on the recent developments of one such microfluidic device nicknamed the mother machine. We overview the original device design, experimental setup, and challenges associated with this platform. We then describe recent methods for analyzing experiments using automated image segmentation and tracking. We further discuss modifications to the experimental setup that allow for time-varying environmental control, replicating batch culture conditions, cell screening based on their dynamic behaviors, and to accommodate a variety of microbial species. Finally, this review highlights the discoveries enabled by this technology in diverse fields, such as cell-size control, genetic mutations, cellular aging, and synthetic biology.

## 1 Introduction

Genetically identical cells can display strikingly different phenotypes within a fixed environment. In multicellular organisms, this is evident through cell differentiation and specialization. Unicellular organisms can also perform specialized behaviors within a group, such as distinct metabolic states (Rosenthal et al., 2018) or discrete roles in biofilm formation (Arnaouteli et al., 2021; Dar et al., 2021). In addition, some species prepare for changing environmental conditions on a population level by keeping a small fraction of the population ready for such changes, a phenomenon referred to as “bet-hedging” (Veening et al., 2008; Morawska et al., 2022). Because biochemical reactions depend on physical interactions between low-abundance molecules, these reactions are inherently stochastic, which results in heterogeneous gene expression between genetically identical cells exposed to the same environment (Elowitz et al., 2002; Paulsson, 2005; Raj and van Oudenaarden, 2008). For these reasons, techniques that can measure single-cell properties rather than population averages have revealed important information about many cellular processes, from cell-size control to differentiation. For example, the ability to measure the mRNA profile of single-cells through techniques such as single-cell RNA-seq (scRNA-seq) has advanced many fields, as evidenced by the breadth and the number of publications in recent years (Karaayvaz et al., 2018; Kinker et al., 2020; Peyrusson et al., 2020). While such techniques have proven to be very useful, they are typically limited to static snapshots and cannot follow gene expression in individual cells over time. Instead, this is typically achieved using single-cell time-lapse fluorescence microscopy, which for microbes has traditionally involved tracking the growth of single bacteria into microcolonies on agar pads. While technically simple and very useful, agar pads only support growth for a short period of time before cells start competing for nutrients, limiting observation to a few cell divisions (Young et al., 2011; Moffitt et al., 2012). Due to limitations in using wide-field fluorescence microscopy (i.e., the point spread function has a long tail), cellular crowding can impact the fluorescence imaging measurements, meaning that the presence of many cells can bias the measured fluorescence of a cell far away (Hardo and Bakshi, 2021). Finally, it can be difficult to change the environmental conditions in this setup, which is important for studying processes such as stress responses. These limitations motivated the development of microfluidic platforms for single-cell analysis that enables single-cell tracking over many generations under precisely controlled conditions.

**FIGURE 1:**
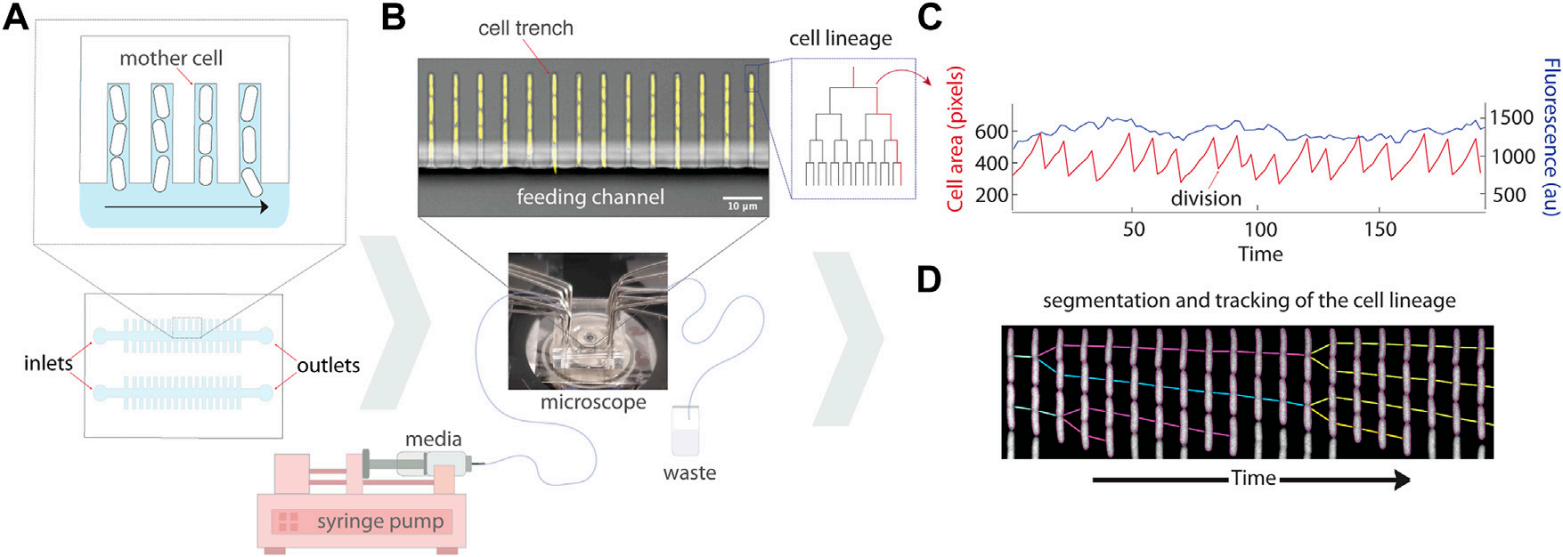
Schematic of the experimental setup for the mother machine microfluidic device and data analysis. **(A)** Schematic representation of the platform which traps single bacterial cells in trenches that are perpendicular to a larger feeding channel. Daughter cells are flushed out of the trenches with flowing media, while mothers remain trapped at the end of the cell trench. **(B)** A micrograph of the mother machine, with YFP fluorescence showing the cells superimposed on a brightfield image of the device. Media is pumped through the inlet into the main feeding channel by a syringe pump, and then exits through the outlet into a waste beaker. **(C)** The lineages of growing cells in the trenches can then be followed under precisely controlled environmental conditions using time-lapse microscopy. **(D)** An example kymograph of a growing cell imaged in fluorescence, illustrating the segmentation and tracking of the lineage.

The first iterations of microfluidic devices for imaging bacteria utilized closed linear trenches (Balaban et al., 2004) or mono-layer chambers (Cookson et al., 2005) to trap cells. The mono-layer chamber devices (Cookson et al., 2005; Ullman et al., 2013) enable the growth of microcolonies while flushing extra cells and continuously providing growth media, and have been particularly useful for studying group behaviors such as quorum sensing (Danino et al., 2010; Prindle et al., 2011; Din et al., 2016; Scott and Hasty, 2016; Miano et al., 2020). Continuous culture devices were then developed to enable long-term imaging of bacterial cells undergoing steady-state growth while facilitating the tracking of single cells without crowding limitations, making them ideal for measuring single-cell gene expression (Wang et al., 2010; Moffitt et al., 2012). A configuration nicknamed the “mother machine” (MM) traps bacteria at the end of single-cell-width dead-end trenches (Wang et al., 2010). Newborn cells are flushed out of the device by the constant flow of growth media, thereby allowing single-cell lineages to be followed for hundreds of generations (Figure 1). The name “mother machine” refers to the fact that the cells trapped at the end of the trenches are tracked growing and dividing throughout an experiment, and are thus referenced as “mother” cells. A similar layout named the chemostat (Moffitt et al., 2012) has trenches open on both sides, thus providing better feeding through convective flow. This device enables long-term time-lapse microscopy while keeping cells that renew both poles and has been used to quantify the maturation time of fluorescent proteins (Balleza et al., 2017).

In this review, we focus on the technical developments of the MM and its applications in the study of bacteria. We start by describing the original design and the constraints that motivated further technical developments. We then overview recent image analysis tools that enable segmentation and tracking of cells in the device using phase contrast images, and modifications to the device that enables the precise control of environmental conditions, screening and isolation of cells, and cultivation of a variety of microbial species. Finally, we highlight how the MM enabled discoveries in a wide range of fields in microbiology, such as cell-size control, genetic mutations, cellular aging, stress responses, cell-fate determination, antibiotic tolerance/persistence, and synthetic biology. For more details, we refer the interested reader to previous reviews that have comprehensively described device fabrication and setup (Weibel et al., 2007; Hol and Dekker, 2014; Eland et al., 2016), discussed challenges associated with single-cell analysis (Yang et al., 2016; Hardo and Bakshi, 2021), and reviewed different microfluidic devices (Weibel et al., 2007; Bennett and Hasty, 2009; Wessel et al., 2013; Vasdekis and Stephanopoulos, 2015; Potvin-Trottier et al., 2018; Scheler et al., 2019).

## 2 Technical developments of the mother machine

### 2.1 Original design and challenges

Performing experiments with the MM requires the following: designing and fabricating a mold for the device, building a microfluidic chip, performing the time-lapse microscopy, and analyzing the images to create time traces. Here, we start with an overview of the original MM design and the challenges that motivated recent technical developments.

### 2.1.1 Device design and fabrication

The original MM design was developed to study *Escherichia coli* cells (Wang et al., 2010). Narrow trenches trap bacterial cells perpendicular to a larger feeding channel that allows media to deliver nutrients to the cells and wash away progeny emerging from the trenches (Figures 1A,B). Several constraints must be considered in the design: the width, height, and length of cell trenches, spacing between cell trenches, and dimensions of the feeding channel. The width and height of the trenches are approximately the dimensions of the particular strain of bacteria being cultivated (e.g., 1.2 μm height, 1.3 μm width, and 20 μm length for *E. coli* MG1655). Proper trench dimensions ensure that the cells are in focus, restrict growth to single file within each trench, and ensure sufficient diffusion of nutrients to all cells in a trench. Too large of dimensions results in cells overlapping each other in the *z* direction–making segmentation and tracking hard to impossible. Too small of dimensions leads to difficulties loading the cells into the trenches and results in poor nutrient diffusion to cells deep in the trenches, including the mother cells. The chosen length of a cell trench is a trade-off between cell retention over time, as short trenches lose cells more rapidly (e.g., through stochastic filamentation that pulls them out in the feeding channel), and feeding of the mother cell. It is thus important to ensure that the growth rate of the mother cell is the same inside the device and in batch culture for each strain and device combination (Yang et al., 2018). Spacing between cell trenches is a trade-off between the throughput (i.e., number of lineages followed per image) and accuracy of fluorescence measurements. Trenches too close to one another can result in biased fluorescence measurements, particularly if neighboring trenches have very different signal intensities (Kaiser et al., 2018; Hardo and Bakshi, 2021). Finally, the width and height of the feeding channel are chosen to minimize hydraulic resistance to facilitate the flow of growth medium, e.g., with syringe pumps. A single centimetre-sized chip can typically fit multiple channels, each with their own inlet and outlet, to enable simultaneous experiments with multiple strains (Figure 1A).

After the design is finalized, a mask can be drawn using CAD software and ordered through different companies (e.g., Toppan Photomasks, United States). The mold is then built on a silicon wafer in a cleanroom environment using photolithography techniques, where the mask is used to expose photo-sensitive resin to light to define the features (Weibel et al., 2007; Eland et al., 2016). The mold is a negative of the features of the chips (i.e., what is solid on the mold becomes air in the device). The ∼1 µm-sized features are on the lower end of the resolution of these techniques, and smaller features (e.g., Section 2.4) require other fabrication techniques (e.g., electron beam lithography). As such, fabrication protocols typically require fine-tuning to obtain the critical feature size (cell trench width and height) within a ∼0.1 µm range, which can affect the experiments as described above. However, once built these molds can be re-used indefinitely to build microfluidic chips. Alternatively, the mold can be ordered custom-built from companies such as: ConScience (Sweden), Cornell NanoScale Science and Technology Facility (United States), Innopsys (France), Kavli Nanolab Delft (Netherlands), Micro Resist Technology (Germany), Sigatec (Switzerland), and TTP (UK). Molds can also be duplicated in epoxy, which can be an inexpensive option for sharing molds between groups (Kamande et al., 2015).

### 2.1.2 Experiment setup

The MM microfluidic chip is made by pouring and curing polydimethylsiloxane (PDMS) on top of the mold, imprinting the features of the mold onto the chip. Individual chips are then cut out of the PDMS slab, and holes are punched (e.g., with biopsy punchers) at the inlets and outlets to allow the connection of tubing which provides growth medium to the device. The chips are then covalently bonded to a coverglass using plasma treatment. Cells can then be loaded into trenches *via* centrifugation with a custom adapter, or by simple diffusion by loading a very dense culture. Tubing is connected to the inlets and outlets (e.g., using syringe needles) for flow of fresh growth media into the chip, as well as removal of used media which has passed through the chip (Figure 1B). The media is typically pushed through the device using syringe pumps, initially at a high flow to clear biofilm that may be growing in dead spaces (i.e., regions of low flow) in the inlets and outlets. The formation of biofilms can be limited by pre-coating the chip with bovine serum albumin (BSA) and/or supplementing the growth media with BSA or Pluronic (Ullman et al., 2013; Cabeen and Losick, 2018). The chip is then mounted on an inverted fluorescence microscope for automated time-lapse microscopy of the lineages growing in the trenches. A cage incubator and hardware autofocus are typically required to ensure stability of the focus over multiple days. Detailed protocols for setting up MM experiments have been published (Cabeen and Losick, 2018). After the experiment, the multi-dimensional images (position, fluorescence channels, time) can be processed to track the properties of single lineages growing in the device (Figure 1C; Section 2.2).

### 2.1.3 Challenges

The original design of the device enabled time-lapse microscopy of single *E. coli* cells under controlled growth conditions, leading to many biological discoveries. There are however some challenges associated with the experimental setup, which have led to new technical developments discussed below. For example, automated image processing traditionally required constitutive expression of a fluorescent protein (FP) at intermediate levels specifically for that purpose. This limited the number of simultaneous reporters that could be used in an experiment, while also preventing the study of bacteria not expressing exogenous FPs. Section 2.2 describes the recent tools developed for segmentation and tracking of cells using phase contrast images. In addition, the original setup of the MM makes switching conditions within an experiment challenging. With this design, growth media can be changed using a Y-junction close to the chip, although this creates a short (and imprecise) delay in the switch as the medium between the Y-junction and the chip is being replaced. Creating complex environmental conditions within the chip, i.e., by flowing a growing culture, can be challenging due to the introduction of air bubbles into the feeding channel. Such challenges have inspired the development of more sophisticated fluidic and environmental control strategies, which are detailed in Section 2.3. Another limitation of the original design is the inability to genotype or isolate cells within the device, which can be useful when imaging libraries of cells. While phenotyping pooled genetic libraries is possible in the device, isolating single cells of interest from the conventional MM platform has been impractical due to the inevitable formation of biofilms in the inlets and outlets, which additionally limit the total duration of an experiment. While cells could potentially be genotyped on-chip through fluorescence *in situ* hybridization (FISH) techniques, diffusion of probes through the cell trenches makes this process inefficient. These limitations have led to the development of techniques for single-cell screening detailed in Section 2.4. Finally, the original device was limited to culturing *E. coli* or other similarly sized microbes. Modifications to trench dimensions and the addition of other features have now allowed the growth and imaging of a variety of other microbes. The cultivation of some species requires additional modifications, which are further described in Section 2.5.

### 2.2 Segmentation and tracking algorithms

While the experimental techniques for using the MM have become increasingly accessible, the image analysis pipeline to convert time-lapse images into single-cell traces through segmentation and tracking has lagged. For many years, laboratories using the MM have developed their own customized data analysis pipelines. While tools have been developed for tracking the growth of microbes on agar pads using phase contrast (Paintdakhi et al., 2016), the large features of the MM PDMS chip can confound such image analysis tools. Recently, multiple open-source software packages have been published specifically for MM experiments with the capability of performing the segmentation and tracking on phase contrast images (listed in Table 1).

**TABLE 1:** Overview of open-source software packages developed for mother machine segmentation and lineage tracking. The applicability to specific imaging modalities indicates which ones have been demonstrated. All programs are available on Github or Gitlab.

Software	Language	Deep learning based	Phase contrast segmentation	Brightfield segmentation	Fluorescence segmentation
Molyso (Sachs et al., 2016)	Python		✓		
MoMA (Kaiser et al., 2018)	Java		✓		✓
MMHelper (Smith et al., 2019)	Python		✓	✓	
BACMANN (Ollion et al., 2019)	Java		✓		✓
DeLTA (Lugagne et al., 2020; O’Connor et al., 2022)	Python	✓	✓		
DistNET (Ollion and Ollion, 2020)	Python	✓	✓		

Most of these image analysis methods share a common overall workflow: pre-processing of the images, segmentation of the cells, and tracking the lineages. In the pre-processing step, the image time series are first registered to correct for drift and jitters of the stage, for example, using cross-correlation between successive images. The images are rotated to align the micro-channels vertically, which simplifies further analyses. The micro-channels are then identified and segmented. Accurately segmenting individual cells is typically the most challenging task, as the cells are small and in contact with each other, and strategies vary between implementations. Tracking is then performed to create time traces of individual cells, where cells from each time point are connected to the cells in the next one, and cell divisions are identified (Figure 1C). Properties such as cell size and fluorescence intensity are extracted along these single-cell time traces. Finally, a manual curation pipeline is typically available, as even rare segmentation and tracking errors can have large effects on sensitive measurements such as the variance.

In Molyso (Sachs et al., 2016), the cell segmentation is done in one dimension with cells identified by bounding rectangles instead of cell contour. The tracking is done by solving an optimization problem, where a cost is imputed for cell displacement between time points and cell division events. MoMA generalized the optimization problem for both the segmentation and tracking, overpredicting possible cell segmentation and performing the tracking simultaneously (Kaiser et al., 2018). This 1D segmentation works well when the cells are perfectly aligned with the channels. However, it can result in errors when the cell width is smaller than the channel width, resulting in tilted cells, or if the cells are not perfectly rod-shaped (e.g., mutants or other bacteria with different morphologies). BACMANN enables 2D segmentation (i.e., cell contour) through watershed-based image processing techniques, while the tracking is based on the position with respect to the top of the trench (Ollion et al., 2019). BACMANN also incorporates a spot-tracking algorithm in its pipeline. MMHelper was developed using similar segmentation approaches to also segment using bright-field instead of phase contrast (Smith et al., 2019). DeLTA (Lugagne et al., 2020) utilizes 3 U-net convolution neural networks (Ronneberger et al., 2015) to perform channel identification, cell segmentation, and tracking. DistNET incorporates a self-attention layer into the U-net architecture to provide information about the whole channel to the neural network, and performs segmentation and tracking through one deep neural network (Ollion and Ollion, 2020). Comparison of BACMANN, DeLTA, and DistNET on the same dataset showed that they could achieve <1% combined segmentation and tracking error rates (Ollion and Ollion, 2020). While BACMANN’s tracking performed better than DeLTA, DeLTA’s segmentation performed better. DistNET’s self-attention layer mainly improved the tracking performance of DeLTA. Benchmarking the techniques against data from different experimental conditions showed good performance but an increased error rate, suggesting a need for more training data or for changing the analysis parameters (Ollion and Ollion, 2020).

When choosing a program, we encourage users to consider the following criteria: accuracy of segmentation and tracking, need for manual curation, speed of analysis, quality of documentation, readability of code, ease of use, flexibility to specific experimental needs, and an actively maintained code-base. Deep-learning methods can be fast and very accurate while requiring training on a relatively small set of manually analyzed data. However, they often lose accuracy when the experimental conditions are different from the training set (i.e., cell size, trench width, *etc.*). Conventional methods typically require changing analysis parameters to accommodate these kinds of changes, but that can be less tedious than manual segmentation of many images required to re-train a deep-learning algorithm. Generating synthetic data to train the neural networks would greatly alleviate their main shortcomings.

### 2.3 Fluidic control and environmental conditions

**FIGURE 2:**
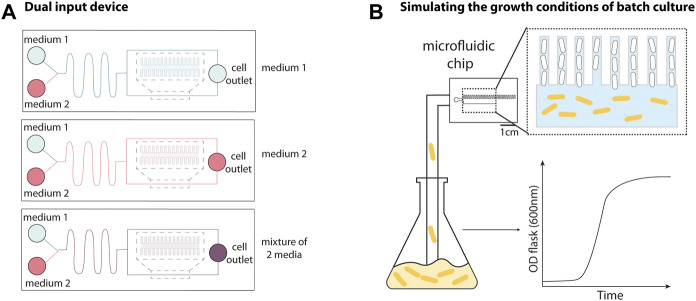
Adaptations to the mother machine architecture for improved fluidic and environmental control. **(A)** The dual input mother machine (DIMM) has two media inlets followed by a serpentine channel that fluid passes through prior to reaching cell trenches to facilitate mixing and/or rapid switching between different environmental conditions. Schematic representation inspired from Kaiser et al. (2018). **(B)** The growth curve platform allows batch culture to be fed into the device to recapitulate batch culture conditions. This allows for observation of cells entering and exiting the stationary phase by switching between nutrient depleted culture and fresh media. Schematic representation inspired from Bakshi et al. (2021).

One key strength of the MM is the precise control over the growth conditions. However, switching between conditions within an experiment and flowing mixed media are challenging using the original design. A rapid and precise switch between growth media can be obtained by modifying the device design to include two inlets for each feeding channel. To flow a mixture of media, it is necessary to introduce a serpentine channel between the inlet and the feeding channel to overcome the mixing limitation of the low Reynolds number environment. The dual input mother machine (DIMM, Figure 2A) utilized this strategy to study the induction of the *lac* operon while switching from glucose to lactose (Kaiser et al., 2018). The authors could track the lag in growth of single cells exposed to this transition and found that the distribution of the growth lag was multi-modal. By quantifying the number of LacY/Z (“sensor”) molecules in single cells in the device, this multi-modality was subsequently attributed to a fraction of the population expressing zero LacY/Z molecules, relying instead on stochastic leaky expression for induction of the operon (Julou et al., 2020). However, any number of expressed lacY/Z molecules was sufficient for fast induction of the operon, making it a single-molecule trigger.

An experimental setup was developed to mimic the conditions of batch culture (Figure 2B), where a growing culture is flowed directly into the microfluidic device, enabling the study of cells transitioning between different growth phases (Bakshi et al., 2021). This was achieved using peristaltic pumps to flow a culture growing in a separate shaker-incubator into the device, and bubble traps to prevent air bubbles from entering the device. By alternating between flowing fresh media and batch culture into the MM, the authors monitored cells after multiple rounds entering and exiting the stationary phase and found that the cell-size regulation strategy changed throughout phases of the growth curve (discussed in Section 3.1).

### 2.4 Screening and isolation based on time-lapse microscopy

Genetic screens have been instrumental in biology for assigning function to molecular components and generally linking genotypes to phenotypes. Many powerful screening platforms have been developed, but they have been mostly limited to distinguishing static phenotypes in the population. Recent technical developments have transformed the MM into a powerful platform that combines dynamic phenotype screening with genotyping or isolation capabilities. This enables screening based on dynamic and/or spatially resolved phenotypes, such as oscillations in gene expression, cell-size control mechanisms, response to changes in environmental conditions, and intracellular localization of proteins. Notably, even static phenotypes could be isolated more precisely because cells can be quantified over many generations, and thus genetic and non-genetic heterogeneity could be distinguished.

**FIGURE 3:**
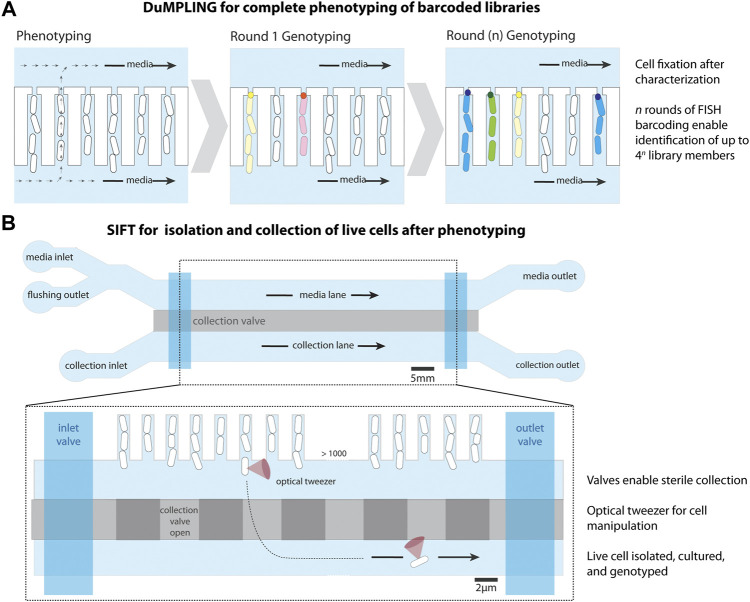
Modifications to the mother machine to enable cell screening. **(A)** Dynamic u-fluidic microscopy-based phenotyping of a library before *in situ* genotyping” (DuMPLING), has a 300 nm gap at the end of the cell trench, allowing media to flow through the cell channels. Rounds of barcoding through FISH enable genotyping of the pooled library. Schematic representation inspired from Lawson et al. (2017). **(B)** Single-cell isolation following time-lapse microscopy (SIFT) uses a modified microfluidic chip containing an additional lane for cell isolation below the cell trenches, separated by a pressurized valve system (collection valve). A second set of valves (inlet and outlet) allows for the lane to be sealed for inlet cleaning and restricting media flow after cell loading. An optical tweezer moves cells of interest from their trench to a collection trap, where they are isolated and removed from the device to be cultured and sequenced. Schematic representation inspired from Luro et al. (2020).

Two techniques have been developed to date for screening cells in the MM. The first technique, named “dynamic µ-fluidic microscopy-based phenotyping of a library before *in situ* genotyping” (DuMPLING) (Lawson et al., 2017), enables dynamic phenotyping of pooled libraries. After characterization of the library in the MM, the cells of the barcoded library are fixed and identified *via* fluorescence *in situ* hybridization (FISH), connecting genotypes to dynamic phenotypes (Figure 3A). A 300 nm gap at the dead-end of the trench is connected to a back-end channel, which generates convective flow that facilitates efficient movement of probes and media over the cells. This convective flow also facilitates feeding of the mother cell therefore reducing the diffusion limitations related to the dimensions of the cell trenches discussed in Section 2.1.1. The DuMPLING platform was used to identify the effects of a CRISPR interference-mediated gene knockdown library on the coordination of replication and division by tracking chromosome replication forks throughout cell division (Camsund et al., 2020).

The second technique, named single-cell isolation following time-lapse microscopy (SIFT) (Luro et al., 2020), uses a modified microfluidic chip containing an additional media channel used for cell isolation (Figure 3B). The device has a system of pressurized valves that separates the cell trenches from the collection channels, temporarily closes the inlets and outlets for sterilization, and closes the media channel to stop the liquid flow. This enables an optical tweezer to move cells of interest from their growth trench to a collection trap where they are isolated, cultured, and sequenced. SIFT was used to screen two libraries of synthetic genetic oscillators based on the periodicity and precision of oscillations, showcasing its strength in isolating dynamic phenotypes.

Both of these screening techniques have advantages and disadvantages. While DuMPLING enables the *in situ* genotyping of entire libraries and allows genotype-phenotype mapping across a large number of cells, it requires a barcoded library and cell fixation prior to hybridization, thereby eliminating any possible downstream growth and analysis (Lawson et al., 2017). Conversely, SIFT does not require barcode labeling, enabling the screening of unmodified libraries and natural populations, the isolation of live cells, and downstream analysis of isolates (Luro et al., 2020). However, only isolated cells can be genotyped, limiting the scale of phenotype-to-genotype mapping throughput. Additionally, the optical trapping mechanism in this technique requires an extensive platform. These extended capabilities for screening and isolating cells within MM-like devices have the potential to enable discoveries in diverse fields of microbiology.

### 2.5 Extension to other microbes

**FIGURE 4:**
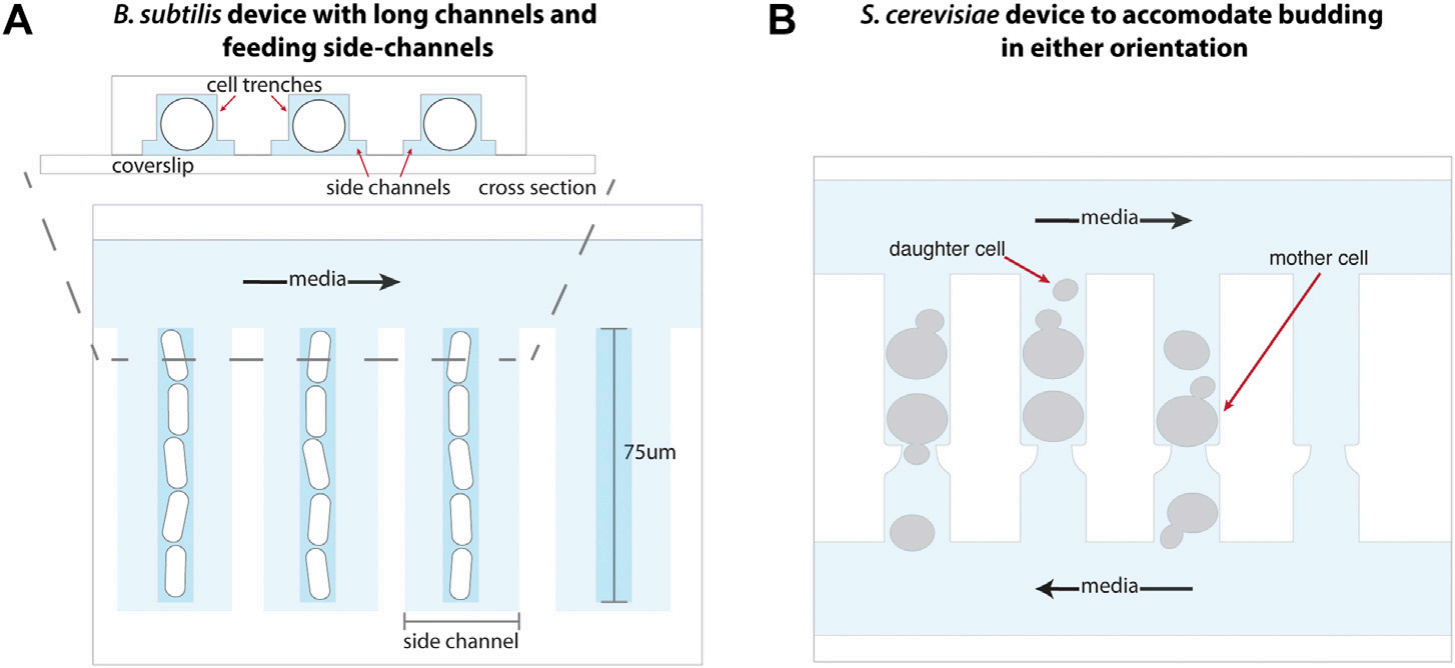
Adaptations to the mother machine architecture to optimize growth of other organisms. **(A)** The mother machine design adapted for *B. subtilis* growth includes elongated trenches 75 μm in length to accommodate its multicellular, chained state, as well as side channels that enable uniform nutrient availability throughout the trenches. Schematic representation inspired from Norman et al. (2013). **(B)** Maintaining an opening at either end of each cell trench in a modified MM device enables removal of *S. cerevisiae* daughter cells produced from budding in either orientation into perpendicular media channels. Schematic representation inspired from Li et al. (2017).

Adaptations to the MM have enabled single-cell studies of a variety of microbes. In principle, adaptation of the MM design to other symmetrically dividing organisms should only be a matter of adapting the trench size, although other minor modifications may be necessary to maintain species-specific optimal growth conditions. Such devices have been fabricated for cultivation of *Corynebacterium glutamicum* (Sachs et al., 2016) and *Bacillus subtilis* (Norman et al., 2013; Cabeen and Losick, 2018). As *B. subtilis* stochastically forms long multicellular chains which would be pulled out of the trenches, an adaptation of the classic MM device incorporated an increased trench length of 75 μm (Norman et al., 2013). This two-layer device included shallower feeding channels surrounding the cell trenches to ensure sufficient feeding of cells at the end of these long trenches (Figure 4A).

The classic MM architecture has been used to study archaea and symmetrically-dividing yeast organisms such as *Halobacterium salinarum* (Darnell et al., 2020) and *Schizosaccharomyces pombe* (Nakaoka and Wakamoto, 2017), respectively, by scaling up device dimensions. The ability of the budding yeast *Saccharomyces cerevisiae* to switch budding orientation over the course of its lifespan necessitated the trapping and retention of mother cells in trenches open on both ends to flowing media (Li et al., 2017). This accommodates the removal of daughter cells produced by budding in either orientation (Figure 4B). Alternatively, the “yeast jail” design forgoes trenches altogether and instead employs a microfluidic device with an array of jail units (Ryley and Pereira-Smith, 2006). Each jail is composed of three PDMS posts (among other designs) that behave as “jail bars” to retain a single mother cell, while daughter cells produced by budding are washed away with the flow of media. Similarly, A Long-term Culturing And TRApping System (ALCATRAS) (Crane et al., 2014), High-throughput Yeast Aging Analysis chip (HYAA) (Jo et al., 2015), and slipstreaming MM (Durán et al., 2020) devices comprise an array of PDMS trapping units, many of which may fit into a single field of view during imaging, to enable tracking of individual mother cells over their lifespan. In the latter, *S. cerevisiae* cells are loaded through the outlet of the device such that media flow reversal enables the trapping of mother cells in a low-pressure zone behind PDMS pillars. This method is ideal for studying cell replicative life span as it does not place cells under mechanical pressure during trapping, and the effect of such stress on aging is not yet known. An alternative method utilizes channels or PDMS structures of optimal heights for trapping mother cells underneath them, while permitting smaller daughter cells to be flushed away (Lee et al., 2012; Xie et al., 2012; Zhang et al., 2012).

## 3 Applications to study bacteria

Several features of the MM have facilitated important discoveries about a variety of cellular processes. The tracking of single cells under controlled and tunable growth conditions with high throughput has shed light on how cellular processes are affected by stochastic gene expression, and how such heterogeneity can affect the cell’s phenotype. The ability to track the same cells for many generations has facilitated studies in cellular aging, and an inherent lack of competition between cells in the device has proven useful in studying genetic mutations. Here we will highlight discoveries about cell-size control mechanisms, genetic mutations, cellular aging, stochastic pulsing of gene expression, phenotypic states in *Bacillus subtilis,* antibiotic resistance and persistence, and synthetic biology that have been enabled by the MM platform. Instead of a detailed description of each study, we highlight how the device has enabled such discoveries.

### 3.1 Cell-size control

Most prokaryotes divide *via* binary fission, yielding two daughter cells of nearly identical size and volume (Angert, 2005). If left unchecked, small fluctuations in cell size at cell division can result in significant size divergence between cells of a population over successive generations. Cell-size homeostasis could in principle be achieved through different control mechanisms (Facchetti et al., 2017). Several studies have shown that *E. coli*, *B. subtilis* and *Caulobacter crescentus* maintain cell-size homeostasis by behaving primarily as “adders”, adding on average a constant length between birth and division, rather than as “sizers” or “timers”, for which cell division is triggered upon cells growing to a threshold size or for a fixed time, respectively (Voorn and Koppes, 1998; Amir, 2014; Campos et al., 2014; Wallden et al., 2016). As time-lapse microscopy on agar pads is limited to a few cell divisions where the growth conditions can change, microfluidic approaches have been important in studying cell-size regulation. The ability to image hundreds of single cells under constant growth conditions while precisely measuring their size throughout the cell cycle (e.g., size at birth and added during the cell cycle) has made the MM a particularly useful platform in elucidating the specific mechanisms underlying cell-size regulation (Taheri-Araghi et al., 2015; Sauls et al., 2019; Si et al., 2019; Witz et al., 2019; Nieto et al., 2020).

Several models have been suggested to explain the mechanism underlying adder behavior (Ho and Amir, 2015; Micali et al., 2018; Si et al., 2019; Witz et al., 2019; Nieto et al., 2020). While a detailed description of the different models is outside the scope of this review, a recent analysis by Le Treut et al. has found the authors’ model, the independent double adder, to be the most consistent with current data (Le Treut et al., 2021). This model proposes that the processes of cell division and initiation of DNA replication are controlled by distinct (independent) adders (Si et al., 2019). It further suggests that the mechanism underlying this type of cell-size control relies on accumulation of specific initiator proteins for independent regulation of cell division and DNA replication to a certain threshold and that they are produced at a rate proportional to cell growth without being actively degraded. This hypothesis was supported by experiments that used the MM to measure the cell size added between cell divisions, and the cell size added between DNA replication initiation events (tracked with DnaN-YPet) (Si et al., 2019), which were both found to be independent of initial cell size (i.e., adders). Perturbing the production of each initiator protein (FtsZ and DnaA respectively) only dysregulated cell-size control for its respective process.

Some studies carried out in the MM have shown deviations from the adder principle under slow growth conditions (Wallden et al., 2016; Si et al., 2019; Nieto et al., 2020). Si et al. (2019) suggest that the adder principle can be broken if the underlying mechanisms (threshold and constant production) are affected. They suggest that active degradation of the division initiatior (FtsZ) could play that role, and elimination of degradation through *clpX* repression restored the adder phenotype under that growth condition (Si et al., 2019). A recent study used a variation of the MM setup which flows batch culture into the device as a means of replicating conditions within that culture on-chip to monitor *E. coli* and *B. subtilis* growth, and revealed that cells alter their size regulation strategy as they progress through different growth phases (Bakshi et al., 2021). Cells switched to mixed “adder-timers” while they entered the stationary phase and behaved as “sizers” while exiting the stationary phase, suggesting that different strategies might be used to respond to changes in environmental conditions. These results also highlight the importance of using the MM for studying this process, as other single cell imaging techniques such as agar pads do not offer precise control over environmental conditions. Developments in the control of growth conditions as well as screening capabilities will thus facilitate the development of more complete models that can capture cell-size control strategies under varying environmental conditions.

### 3.2 Genetic mutations

Mutations and other DNA damage events such as double-stranded breaks (DSBs) have the potential to drastically affect cell survival and fitness if left unrepaired (Gordo et al., 2011). To counter such mutations, *E. coli* employs effective DNA repair pathways to recognize and repair genetic perturbations before they are propagated to future generations. The ability to track thousands of lineages in parallel (without growth competition between individuals) makes the MM an ideal platform to study rare events such as the emergence of DNA mutations. Its capacity to capture heterogeneity in number and types of mutations emerging between isogenic cells of a population further highlights the utility of such single-cell, time-lapse studies. To this end, the MM has been used to visualize copy number variations (Tomanek et al., 2020), DNA mismatch error (Robert et al., 2018, 2019), DSBs (Wiktor et al., 2021), and alkylation damage (Uphoff et al., 2016; Uphoff, 2018; Vincent and Uphoff, 2021).

The timing of mutations as well as their impact on the fitness of the cells are important variables in the process of evolution. The rate of chromosomal gene copy number mutations in *E. coli* was estimated at ∼3 × 10^−3^ per cell per generation in the MM (Tomanek et al., 2020). This was achieved by expressing several copies of a chromosomally integrated fluorescent reporter and measuring changes in fluorescence over time as an indicator for gene copy number (because gene expression is roughly proportional to gene copy number). Robert et al. leveraged the lack of competition in the MM to visualize the appearance of DNA replication errors and their fitness impact (Robert et al., 2018, 2019). To measure the fitness effect of the mutations, they measured the decrease in growth rate (i.e., fitness) of the individual lineages through the accumulation of mutations (Robert et al., 2018). Instantaneous single-cell growth rate can be calculated in the MM using the relative change in cell size between successive time points (i.e., doubling rate). Although the entire distribution of fitness effects (DFE, which has proven challenging to measure in the field of evolution modeling) was still not directly estimated, the authors were successfully able to infer all the moments of DFE (i.e. average, variance, skewness, etc.) - thanks to the ample statistics enabled by the MM. The analysis indicated the underlying distribution to be long-tailed, with most mutations having little-to-no cost on cell fitness: mean fitness cost was only ∼0.3 %, which was apparently overestimated by the previous studies. In contrast, 1% of the mutations were found to be lethal, directly measured by observing cell death in the MM. Mutations were detected by tracking the appearance of YFP-MutL foci, and occurred at a constant rate over time during steady state exponential growth, as expected from a memoryless Poisson process. However, another MM study showed that a subset of cells within a population exhibited a period of elevated mutation rates in response to DNA alkylation damage, due to delayed activation of the Ada DNA damage response regulon (Uphoff, 2018; Vincent and Uphoff, 2021). These periods of high mutation rate lasted several generations, since many cells had no sensor-activator Ada molecules present, and had to wait for stochastic expression to induce the response (Uphoff et al., 2016). Finally, the response to DSB has also been characterized using an adapted MM with multiple inlet ports connected to the feeding channel *via* a short junction, enabling rapid switching between different media (Wiktor et al., 2021). Switching between a growth medium and an induction medium enabled short induction of Cas9, which created a targeted DSB in the chromosome. The chromosomes could be tracked using fluorescently-tagged proteins (ParB and MalI) with DNA-binding sites localized close to the DSB, with the foci disappearing during the DSB. Repairs were rapid (∼15 min), homogeneous, robust (∼95% of cells repaired the damage), and had low impact on the fitness of the cells. This is impressive given that the DSB needs to find/colocalize with its repair template on the sister chromosome. The RecA-single stranded DNA complex was observed to extend in a long filament spanning the length of the cell. This could facilitate the homology search for the repair template by eliminating the need to search along the length of the cell in the *z* direction, thereby reducing it from a 3D problem to a 2D one and making the search process 100 times faster.

### 3.3 Aging

The phenomenon of aging in unicellular organisms is broadly described in terms of senescence, or the progressive loss of fitness over time (Moger-Reischer and Lennon, 2019). This loss of fitness can be due to a decrease in growth rate and/or an increase in death rate. Defining the ‘age’ of a unicellular organism that lacks replicative asymmetry can be challenging, but generally takes into account the asymmetric segregation of damage factors during cell division, which creates effectively ‘older’ and ‘younger’ progeny cells. For example, in budding yeast asymmetric division creates a finite replicative lifespan by partitioning detrimental cellular factors such as misfolded protein aggregates to older ‘mother’ cells, while preserving the daughter lineage (Knorre et al., 2018). The mother exhibits senescence over successive generations and eventual cell death. While *E. coli* divides symmetrically, there is still an intrinsic asymmetry in the process: one pole is created during division (the ‘new’ pole), and one is left intact (the ‘old’ pole) (Proenca et al., 2018). Asymmetries in partitioning of cellular contents have also been observed in *E. coli*. For example, the main efflux pump (AcrAB-TolC) was shown to be partitioned with a bias for the old pole cell, leading to elevated efflux activity in ‘older’ cells (Bergmiller et al., 2017). Non-random segregation of sister chromatids has also been observed, with the ancestral strand being partitioned preferentially in the old pole cell (Mäkelä et al., 2021). The MM is uniquely suited to study aging in symmetrically dividing bacteria as it retains an old pole cell at the end of each trench over an entire experiment, thus facilitating monitoring of the old pole lineage for many generations. Here, we present findings from several studies monitoring the aging process in *E. coli* in the MM, where the age of a cell is defined as the age of the old pole.

Both decreased growth rate and increased death rates have been associated with aging in *E. coli* (Lindner et al., 2008; Wang et al., 2010; Proenca et al., 2019; Łapińska et al., 2019). The first report of the MM studied the aging of the mother cell over consecutive generations (Wang et al., 2010). They found that the growth rate of the mother cell was stable for more than a hundred generations, but observed that the filamentation rate increased until 50 generations (Wang et al., 2010). Note that in 50 generations of exponential growth, 1 cell would divide into ∼10^15^ cells, making such studies intractable without the use of devices like the MM. Subsequent studies tracked the growth rate of both the mother cell and its immediate progeny, the ‘daughter’ cell. This showed that these cells reached different equilibrium growth rates, with the mother cell stabilizing at a growth rate slightly slower than the daughter cell (Proenca et al., 2018, 2019). This suggests that damaged molecules divided asymmetrically, with a preference to the old pole cell. The nature of this damage and the mechanisms underlying asymmetrical partitioning of cellular components are under investigation. Misfolded proteins are a prime suspect, as a chaperone fusion (IbpA-YFP) was shown to preferentially localize to the old pole as a foci (Lindner et al., 2008; Proenca et al., 2019), while protein stress such as phototoxic stress affected the asymmetry (Proenca et al., 2019). However, FP fusions such as YFP have been shown to create artifactual foci due to their oligomeric properties (Landgraf et al., 2012). Therefore, the use of FP fusions that do not cause aberrant foci (e.g., ClpB-msfGFP) will be informative in tracking protein aggregates (Govers et al., 2018). In contrast, another study has shown no asymmetries in misfolded protein aggregates using brightfield imaging for inclusion bodies and ThT dyes to visualize protein aggregates (Łapińska et al., 2019). Asymmetric retention of protein aggregates at the old pole has also been observed in the symmetrically dividing fission yeast *S. pombe* in a MM-like device (Nakaoka and Wakamoto, 2017). These aggregates were not associated with increased division times and were eventually transferred to a new daughter, thereby rejuvenating the old lineage. While it is clear that even cells dividing “almost symmetrically” like *E. coli* exhibit aging, the elucidation of the molecular mechanisms and the agents causing this phenomenon will shed light on the universal properties of cellular senescence, and the MM provides an ideal platform with which to study it.

### 3.4 Stochastic pulsing

Stochastic fluctuations in gene expression can drive phenotypic heterogeneity among clonal bacterial populations, such that genetically identical cells display distinct behaviors. The use of FP reporters for gene expression have been invaluable in quantifying this heterogeneity. In principle, this heterogeneity can be generated on different timescales. For example, cells can stochastically express genes at different levels for long periods of time (e.g., stable epigenetic state), or they can fluctuate rapidly between these different expression levels (e.g., rapid pulses). Long-term time-lapse microscopy using microfluidic devices such as the MM enables tracking the expression dynamics in thousands of individual cells under controlled growth conditions and can elucidate the timescales of such fluctuations. Employing this strategy, it was shown that the promoters controlling flagellar biosynthesis genes in *E. coli* activate in stochastic pulses even if expression of their master regulator was constant (Kim et al., 2020). Measuring expression of a gene (*bolA*) dependent on the *E. coli* general stress response factor, RpoS, revealed heterogeneous expression in liquid culture, in the MM, and in another microfluidic device (Patange et al., 2018). The promoter activity (production rate of the reporter) can be calculated using the derivative of the fluorescence intensity while accounting for dilution of the FP present (Locke et al., 2011). The promoter activity revealed stochastic pulses of expression which coincided with periods of slower growth in the device (Patange et al., 2018). The ability to rapidly change environmental conditions and measure single-cell properties prior to and after the change enabled them to test whether these periods of slow growth led to increased resistance to stress. They observed that cells with higher RpoS activity and slower growth immediately prior to the stress were more likely to survive a hydrogen peroxide treatment. Another study measured the expression of multiple stress response genes in the MM and found additional RpoS-dependent promoters exhibiting stochastic pulses that negatively correlated with growth rate (Sampaio et al., 2022). Genes from the SOS regulon also displayed pulsatile activity, but these were not correlated with the growth rate. Cells undergoing pulses of genes from both these groups prior to a short treatment of the antibiotic ciprofloxacin in the MM had increased likelihood of survival (Sampaio et al., 2022). Pulses in the SOS regulon have also been observed in another study using the MM, and have been attributed to variability in the degradation of its regulator, LexA (Jones and Uphoff, 2021).

In *B. subtilis*, several sigma factors have been reported to exhibit stochastic, pulsatile bursts of expression (Locke et al., 2011; Cabeen et al., 2017; Park et al., 2018), which can be similarly tracked in the MM. Sigma factors in *B. subtilis* promote the production of their own operons, which also encodes their anti-sigma factors, creating positive and negative feedback that can cause pulses of gene expression. Molecular “time-sharing” was proposed as a mechanism in which alternative sigma factors competing for RNA polymerase binding opportunities are able to share such core resources over time (Park et al., 2018). Stochastic bursts of sigma factor activity also seem to play a role in stress response in *B. subtilis*: heterogeneous response to lysozyme stress was observed between individual cells grown in the MM (Schwall et al., 2021). Pulsatile expression of the *sigV* operon preceding exposure to lysozyme stress led to increased survival probability.

### 3.5 Phenotypic states in *B. subtilis*

Early work with *B. subtilis* in the MM revealed the existence of a cell fate switch controlling whether *B. subtilis* exists in a free-living, motile state or as a sessile member of a multicellular chain associated with biofilm formation (Norman et al., 2013). A modified device was used, with side-channels to ensure even nutrient availability in the long channels that accommodate the chain phenotype. By tracking the fates using fluorescent reporters over hundreds of cell generations, it was found that the transition from the motile to the sessile state happened at a constant rate over time (i.e., is a memoryless process), but the cells spent a precise amount of time in the sessile state. A simple network of three proteins could recapitulate all the properties and the modularity of the switch and the commitment to the chained state. Remarkably, the circuit was reconstituted in evolutionarily distant *E. coli*, showing that this simple network is sufficient to drive cell-fate decision making (Lord et al., 2019). The stochastic entry into another cell fate–sporulation–was also studied using the MM, and was shown to occur at a constant rate over time after adaptation to the sporulation-inducing conditions (Russell et al., 2017).

In another study, a clonal *B. subtilis* population diverged into subpopulations of distinct metabolic specialists, each characterized by differential expression of metabolic genes (Rosenthal et al., 2018). Cells with stochastically upregulated *sucC* expression in mid to late exponential phase were associated with the production of acetate, while a subpopulation expressing *alsS* in early stationary phase was linked to production of acetoin. Cells could be observed stochastically switching in and out of such states with fluorescent reporters in the MM. As acetoin can neutralize low pH conditions caused by acetate accumulation, the slow-growing *alsS*-expressing subpopulation enabled growth and expansion of an *alsS-* subpopulation which benefited from the neutralization of acetate in an agar pad microenvironment. This showcases how stochastic gene expression can help populations of genetically identical cells achieve cooperative behaviors. Therefore, bacteria have shown the ability to harness molecular fluctuations through simple circuits of a handful of proteins to establish heterogeneous phenotypic states that can be advantageous to the bulk isogenic population.

### 3.6 Antibiotic resistance and persistence

The rise of antibiotic resistance combined with the lack of new antibiotics is an alarming threat to public health (Aslam et al., 2018). Persister cells can survive antibiotic treatment by remaining in a temporary state of dormancy throughout antibiotic exposure, without being genetically resistant (Balaban et al., 2019). The switch to this state can happen spontaneously or be induced by stress such as starvation. Microfluidic devices are particularly well-suited for the study of this non-genetic heterogeneity since growth conditions can be precisely controlled and single lineages tracked over time. One of the first applications of a MM-like device was to establish the persister state as a phenotypic switch (Balaban et al., 2004). Persisters can be identified in such microfluidic devices as cells that are not growing prior to antibiotic exposure, but resume growth at some later time point following removal of the antibiotic. With an increase in throughput of the MM and microscopy (e.g., more trenches per chip and faster microscope imaging with larger field of view), it was possible to observe hundreds of *E. coli* persisters without mutations that increase their typically low frequency of approximately 1 in 1000 cells (Bakshi et al., 2021). The molecular mechanisms underlying this phenotypic switch are still under investigation (Goode et al., 2021b; Manuse et al., 2021). Studies using the MM enabled the characterization of these persisters and have shown that they have smaller size (Bakshi et al., 2021), lower ATP levels (Manuse et al., 2021), and are more likely to contain protein aggregates (Goode et al., 2021a). Recent developments in microscopy throughput, simulating batch culture conditions, and screening in the MM will likely help us to understand the molecular mechanisms behind bacterial persisters.

The MM has also been used to study more broadly the response of bacteria to antibiotics. The device with back-channels (Figure 3A) was used to rapidly load cells into the device for fast antibiotic susceptibility testing (∼30 min) of clinical samples (Baltekin et al., 2017), by directly visualizing growth or death of the bacteria during antibiotic exposure through microscopy. A study looked at the accumulation of the antibiotic ofloxacin inside *E. coli* in the MM and has shown that stationary phase cells appeared to absorb the antibiotic more slowly than exponentially growing cells (Cama et al., 2020).

### 3.7 Synthetic biology

It has become increasingly clear that cellular circuits must contend with stochastic gene expression and that this noise can have an important impact. Therefore, it is valuable to have the ability to quantify such variability for the engineering of cells for synthetic biology applications. Indeed, microfluidic devices have been instrumental in the development of synthetic gene circuits with dynamic properties (Cookson et al., 2005; Stricker et al., 2008; Danino et al., 2010; Din et al., 2016; Lezia et al., 2022). The use of the MM and insights from theory of stochastic gene expression enabled the re-engineering of the repressilator - the iconic synthetic oscillator that helped kick-start the field of synthetic biology—to achieve a precision that approaches natural oscillators (Potvin-Trottier et al., 2016; Luro et al., 2020). The MM was instrumental in enabling the precise characterization of the oscillators, identifying factors that disrupted oscillations, characterizing redesigned iterations of the circuit, and subsequent screening of pooled libraries. While a handful of studies have used the MM to evaluate (Niederholtmeyer et al., 2015; Zhang et al., 2022) or control (Lugagne et al., 2017) synthetic gene circuits, the broader use of the MM throughout the design process could lead to a new generation of precise and robust synthetic circuits.

## 4 Discussion

Single-cell microfluidic platforms have facilitated important discoveries in a variety of fields in biology by enabling the quantification of dynamic and heterogenous processes. Recent technical developments of the MM, including the ability to phenotype or screen pooled libraries based on their dynamics, achieve better control over the growth conditions, and to culture additional organisms should continue to expand the applications of these devices to new fields. Further developments could facilitate the study of species-species interactions, as has been done in a few studies (Din et al., 2016; Cooper et al., 2017). The majority of studies using the MM have focused on *E. coli* and *B. subtilis*, and applications of the device to other organisms (e.g., microbes important in the clinic or in the gut microbiota) would broaden its scope. Outside the microbial realm, two studies so far have used the device with non-adherent mammalian cells (Pearl Mizrahi et al., 2016; Seita et al., 2021). More studies could shed light on dynamic and heterogeneous processes, such as phenotypic resistance to cancer treatment and the differentiation of hematopoietic stem cells (Huang, 2009; Jolly et al., 2018; Reyes and Lahav, 2018). Adaptation of the phenotyping and screening platforms to mammalian cells could provide an alternative to the single-cell screening techniques that have been developed (Stuart and Satija, 2019; Chandrasekaran et al., 2020).

Future studies using the MM will likely continue to advance studies of the heterogeneous processes discussed above. By showcasing discoveries in diverse fields, we hope to inspire the readers to implement such devices to explore questions in new fields and expand their possible applications. For example, the timing of other epigenetic processes in bacteria, such as prions (Yuan and Hochschild, 2017; Fleming et al., 2019), could be elucidated. While many studies have quantified the heterogeneity in protein production, few studies have examined how the degradation of proteins can create heterogeneity (Wong et al., 2007; Jones and Uphoff, 2021). Although most bacteria in nature are in stationary phase (Gefen et al., 2014), the majority of studies have focused on exponentially-growing bacteria. The technical developments of the MM enabling the observation of cells in different growth phases could fill this gap in the literature and generate insights into the stationary phase. Adopting the MM setup has become increasingly accessible, with molds available through different companies, detailed protocols published, and open-source data analysis software now available. Ultimately, because these microfluidic devices provide a new quantitative way to look at cells, they have the potential to continue to contribute to discoveries in diverse areas in biology.
